# Influence of Silica Fume on High-Calcium Fly Ash Expansion during Hydration

**DOI:** 10.3390/ma15103544

**Published:** 2022-05-15

**Authors:** Yurii Barabanshchikov, Kseniia Usanova

**Affiliations:** Institute of Civil Engineering, Peter the Great St. Petersburg Polytechnic University, 195251 St. Petersburg, Russia; ugb@mail.ru

**Keywords:** fly ash, cement, binder, concrete, silica fume, microsilica, expansion, heat of hydration, X-ray diffraction analysis, differential thermal analysis

## Abstract

The purpose of this work was to study the possibility of neutralizing high-calcium fly ash expansion during hydration. The object of the study was the fly ash of Berezovskaya GRES, which is capable of independent setting and hardening. The test in the Le Chatelier molds showed that the divergence of indicator arms was 90–100 mm 1 day after mixing with water. The expansion and cracking of the fly ash could be completely prevented by silica fume addition in an amount of 42.9% by weight of the fly ash. At the same time, the compressive strength of specimens from the fly ash–sand paste in a ratio of 1:5 at the age of 28 days was 1.47 MPa. The isothermal heat release at a temperature of 20 °C for 10 days reached 500 kJ/kg. XRF and DTA results showed that free lime in the fly ash was completely hydrated in 11 days and gave the greatest expansion in the absence of silica fume. The presence of silica fume made the lime hydration incomplete and decreased the expansion. Unslaked free lime remained in the system. Exothermic data showed that silica fume inhibited CaO hydration from the reaction start.

## 1. Introduction

Fly ash is an industrial waste generated from the combustion of solid fuels in thermal power plants, and it is captured by electrostatic precipitators or bag filters before the flue gases are emitted [1]. The main areas of fly ash use are road construction and soil stabilization [2,3,4], as well as production of concrete [5] and geopolymers [6,7]. In concrete production, fly ash can be used as an additive [8,9], as a partial replacement of cement [10,11,12], or as coarse aggregate in granular form [13,14]. The chemical composition, physical properties, mineralogical composition, and morphology of fly ash are very important when used in these areas [15,16].

The economic effect of the use of industrial waste such as fly ash or slag in the production of mortars and concrete is to save cement or natural aggregates [17,18]. This method of disposal of fly ash as industrial waste is dictated not only by economic considerations, but also by environmental protection requirements [19,20,21]. Fly ash dumps contribute to pollution of the air and water basins, as well as changes in the chemical and mineral composition of soils [22,23]. Dispersal of fly ash dumps pollutes the environment and adversely affects human health. Water filtration in the fly ash dump changes the natural hydrochemical regime in the zone of its location, which can lead to flooding, salinization and swamping of the territory, and the entry of pollutants into groundwater.

In the production of concrete, there are restrictions on the use of fly ash, associated with the content of free calcium oxide (CaO_free_). CaO_free_ constitutes particles covered with a vitreous shell, which is difficult to reach for contact with water in the initial stages of interaction. This circumstance leads to hydration of calcium oxide at a later age, when the bulk of the material has already hardened and can crack when CaO transforms into Ca(OH)_2_, accompanied by an increase in volume. The high content of CaO_free_ (>10%), leading to strong expansion and cracking during the curing period, is a serious problem that prevents the use of high-calcium fly ash in concrete technology [24,25,26].

The authors of [27] defined additives that minimize the expansion and appearance of cracks on specimens made from Portland cement with partial replacement with high-calcium fly ash in the range of 30–70% of the total cementitious material, by mass. Superplasticizer in the amount of 0.5–1.0% reduced heat of hydration and simultaneously reduced the expansion and minimized the crack appearance. The addition of silica fume in the mixture caused shrinkage that minimized the expansion, thus lowering the crack development. The influence of silica content on the concrete expansion was also confirmed in [28,29,30].

The authors of [31] reported that blended cements of type Cem IV/B with as much as 50% fly ash could be produced without any risk for expansion or other failure. To do this, fly ash with initial CaO_free_ content of up to 16% required grinding, while fly ash with a high CaO_free_ content required both grinding and hydrolysis. Simultaneous grinding and hydrolysis of fly ash for the reduction of CaO_free_ was also proposed in [32].

Different types of fibers influence the expansion control of the paste consisting of cement and high-calcium fly ash with a high content of CaO_free_, e.g., carbon fiber [33] and alkali-resistant glass fiber [34,35].

However, not much research exists on the neutralization of the destructive potential of a cement-free binder consisting only of high-calcium fly ash. Pre-hydration of fly ash to reduce both ettringite formation and expansion was investigated in [36,37]. It was shown that the high lime content of the sulfocalcic component in the blends transformed into portlandite. Higher mechanical strength was achieved without disintegration of the mortar [37].

The authors of [38] proposed using cavitation technology. The experiments demonstrated that the cavitation phenomenon resulting in the formation and collapse of cavities after a high-rate run through the system of special obstacles was highly effective for hydrodynamic dispersion and slaking of the CaO_free_ excess in brown coal ashes. When using fly ash containing 15% CaO_free_, fly ash paste expansion was reduced by 50% under the optimum parameters of the ultrasound treatment.

The authors of [39] demonstrated that grinding is beneficial to improve the volume stability property of hardened fly ash paste. This is because grinding of the fly ash can destroy the sulfate shell generated during SO_2_ removal, as well as decrease the particle size, which quickens the hydration of anhydrite and lime. 

The methods described above complicate the production of a cementless binder based on high-calcium fly ash with a high content of CaO_free_. An easier way to neutralize the fly ash expansion would greatly expand its use in concrete technology, as an alternative to Portland cement, as a coarse aggregate after granulation, or as an expansion agent, as described in [40].

For the experimental part of the work, fly ash from Berezovskaya GRES was chosen. This fly ash is characterized by a high content of CaO and CaO_free_.

The aim of the study was to neutralize the expansion of high-calcium fly ash from Berezovskaya GRES during its hydration for further granulation without the use of binders.

## 2. Materials and Methods

### 2.1. Materials

Fly ash is produced at Berezovskaya GRES. The Berezovskaya GRES Branch of PJSC Unipro is located in the Sharypovo district of the Krasnoyarsk Territory (Russia). The plant is operated using lignites from the Kansk-Achinsk coal basin. The installed capacity is 2400 MW.

Fly ash from coals of the Berezovsky deposit is mineralogically represented mainly by silicates, aluminosilicates, and calcium ferrites, as well as calcium and magnesium oxides. Small amounts of TiO_2_, MnO_2_, and P_2_O_5_ are also present. Fly ash is hydration-active; it reacts with water and hardens.

The chemical composition of fly ash from the coal combustion of the Berezovsky deposit is characterized by a high content of CaO_free_ (10–30%). The average composition of fly ash is presented in Table 1.

The physical properties of fly ash are presented in Table 2.

The physicochemical characterization of the fly ash (Table 1 and Table 2) was provided by the supplier. Additional tests were carried out, including X-ray diffraction analysis. 

Table 3 shows the identified phases in the tested fly ash sample. 

**Figure 1:**
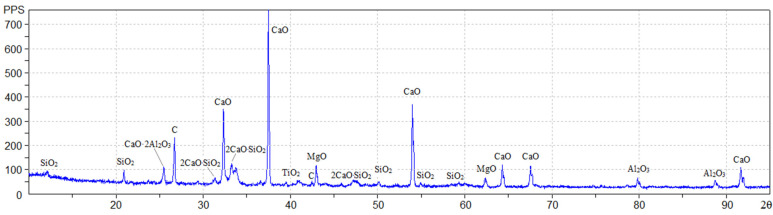
X-ray diffraction pattern of fly ash from Berezovskaya GRES.

The corresponding X-ray diffraction pattern is shown in Figure 1.

### 2.2. Fly Ash Expansion Test in Le Chatelier Molds

**Figure 2:**
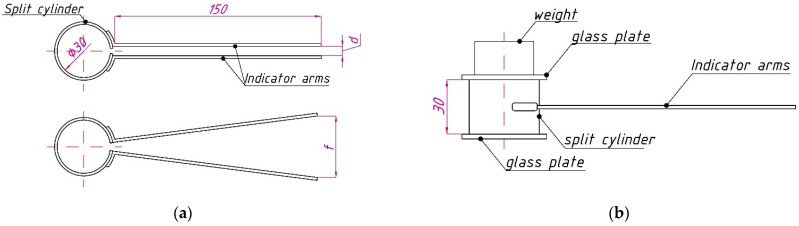
Top view before and after specimen expansion (**a**) and side view (**b**) of Le Chatelier mold.

Le Chatelier molds were used for the expansion test (see Figure 2). Fly ash in the amount of 50 g and additive in the amount indicated in Table 4 were required to fill one Le Chatelier mold. First, the dry mix of fly ash and additive was mixed manually, and then water was added in small portions until all compositions had approximately the same consistency, which was evaluated visually and by the mixing force. Fly ash expansion tests were carried out according to the method of EN 196-3:2016 “Methods of testing cement part 3: determination of setting times and soundness”. The Le Chatelier molds were placed on glass plates and filled in one step with fly ash paste without compaction. The excess paste was cut off with a knife. The molds were covered on top with plates with a weight of 100 g and cured in air at a temperature of 20 ± 2 °C and relative air humidity of 45–55%. In contrast to EN 196-3:2016, the specimens were not boiled. The distance *f* between the ends of indicator arms was periodically measured with a caliper with an accuracy of 0.5 mm, and the difference Δ*f = f − d* was calculated, where *d* is the distance before the experiment.

The types of additives used and their content in the paste by fly ash mass are presented in Table 4. 

### 2.3. X-ray Diffraction Analysis and Differential Thermal Analysis

The use of X-ray diffraction analysis (XRD) and differential thermal analysis (DTA) was aimed at establishing what interactions occur in the fly ash–silica fume system and how they are related to expansion. The analyses were performed on hardened fly ash paste previously tested in Le Chatelier molds (specimens 11–16 in Table 2). Specimen preparation for XRF and DTA included grinding and vacuum drying for 3 h at a residual pressure of 3.3 Pa. The dried specimens were ground in an agate mortar until they passed through a sieve with mesh 0.05 mm.

Semiquantitative analysis of crystalline phases in the specimens was carried out on a Dron 7 X-ray diffractometer produced by JSC “EC” Burevestnik (Russia) with the following parameters: CuKα radiation, λ = 0.15406 Å, 2θ shooting range from 8° to 94° with a step of 0.02°, and an exposure of 3 and 5 s.

Differential thermal analysis was performed on the device “Termoscan-2” produced by LLC “Analitpribor” (Russia). The specimens for DTA had a mass of about 0.7–0.8 g. The specimens were heated to a temperature of 950–1000 °C.

### 2.4. Heat of Hydration by Means of Semi-Adiabatic Calorimetry

#### 2.4.1. Determination Method

The heat of hydration of the fly ash–sand mortar was determined experimentally using semi-adiabatic calorimetry according to EN 196-9:2010 “Methods of testing cement part 9: heat of hydration—semi-adiabatic method”.

**Figure 3:**
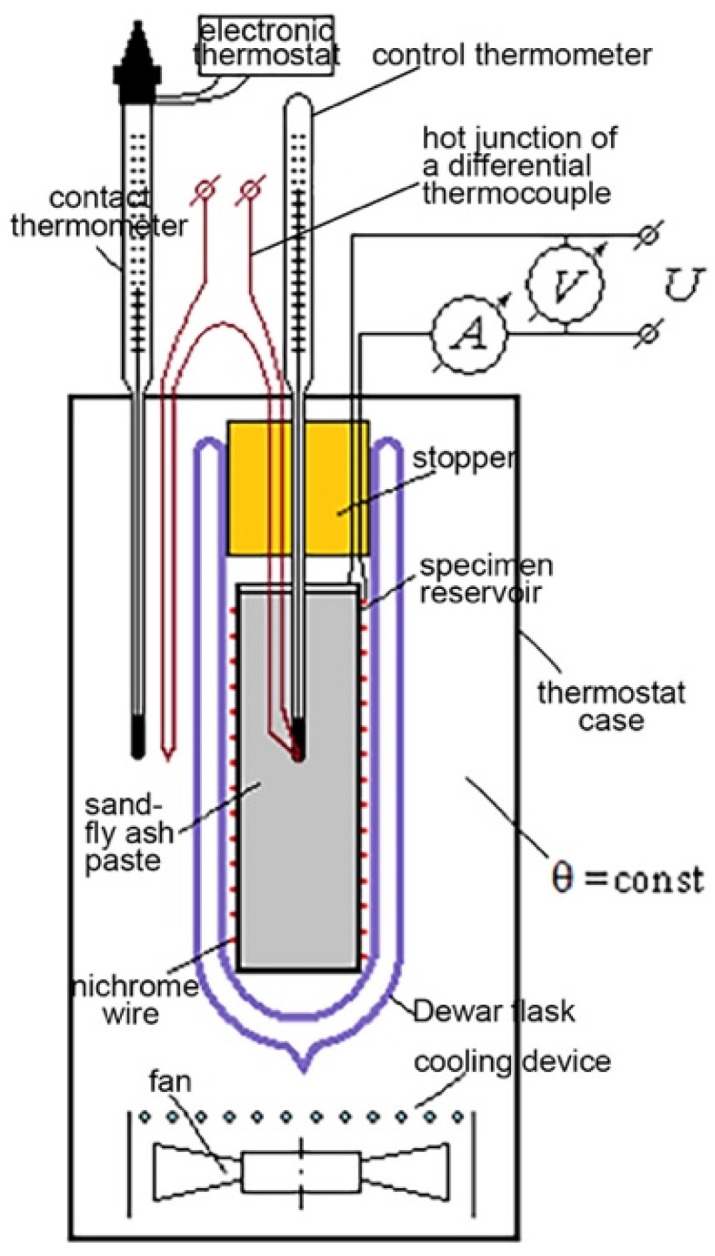
Calorimeter scheme.

The scheme of the calorimeter manufactured in the laboratory of Peter the Great St. Petersburg Polytechnic University is shown in Figure 3. The fly ash–sand paste was placed in a thin-walled aluminum container and placed in a glass Dewar flask. A hot junction of a differential thermocouple and a control thermometer were placed in the center of the specimen reservoir. To determine the heat capacity, the specimen was supplied with an electric heater in the form of an insulated nichrome wire wound around the cylindrical surface of the specimen reservoir.

The Dewar flask with a specimen, closed with a polystyrene foam stopper, with heater and thermocouple wires, was placed in the center of a heat-insulated thermostat case. To regulate the air temperature in the thermostat, it was equipped with a cooling device and a fan. During the experiments, the temperature of the air environment *t_e_* surrounding the thermos was maintained constant using a contact thermometer and an electronic thermostat. Fluctuations in the temperature of the medium relative to the average value were ±0.3 °C. Specimen temperature was recorded automatically every 30 min with an accuracy of 0.1 °C.

#### 2.4.2. Heat of Hydration Determination 

When measuring the exotherm of the specimen, the heating element was not used or was absent.

The heat *Q* released by the specimen during the hydration of the fly ash was used to heat the contents in the Dewar flask (*Q*_1_) and was lost to the environment (*Q*_2_).

*Q* = *Q*_1_ + *Q*_2_. (1)

The amount of heat *Q*_1_ is defined as

*Q*_1_ = *C*(*t* − *t*_o_), (2)

where *C* is the additive heat capacity of the Dewar flask contents (J/°C), i.e., the specimen, aluminum container, heating element, ends of the thermometer and thermocouple, part of the polystyrene foam stopper, and the inner part of the Dewar flask; *t*_o_ is initial specimen temperature, and *t* is specimen temperature at time *τ*. 

The additive heat capacity was determined experimentally as described in the next section.

The amount of heat lost by the specimen due to the convective heat exchange of the Dewar flask with the surrounding air was calculated using Newton’s formula.

(3) $$Q_{2}=\alpha S\int_{0}^{\tau }\left(t-\theta \right)d\tau ,$$

where α*S* is the Dewar flask heat transfer constant (the amount of heat lost or gained by a Dewar flask in 1 s at a temperature difference between the specimen and the medium of 1 K), α is the heat transfer coefficient, *S* is the body surface area, θ is the medium temperature (maintained constant), and *τ* is the time. The Dewar flask heat transfer constant was determined experimentally.

The integration was replaced by summation over the area enclosed between the curve of the specimen temperature versus time *t = f*(*τ*)** and the straight line corresponding to the constant temperature of the medium θ = *const.*

#### 2.4.3. Additive Heat Capacity Determination

**Figure 4:**
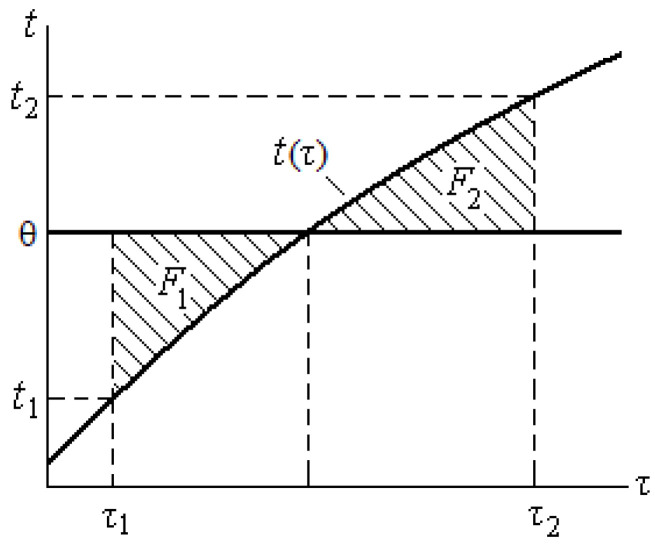
Graph for determining the temperature interval for heating the specimen.

After the end of the experiment, to determine the heat of hydration, the specimen was heated and kept at a temperature of 60 °C for 2 days to complete the possible physicochemical processes. Then, the specimen was removed and cooled to a temperature of 18–20 °C below the ambient temperature θ. After installing the cooled specimen in the calorimeter, it was slowly heated over 1.5–2 days to a temperature 20–22 °C higher than the ambient temperature θ, applying a stabilized voltage (*U = const*) of several volts to the specimen heater (see Figure 4). At the same time, the temperature of the specimen *t*, current strength *I*, and voltage *U* were recorded. Slow heating was necessary to equalize the temperature inside the Dewar flask. Due to the high thermal resistance of the Dewar flask and the polystyrene foam stopper, at very slow heating, the temperature inside the Dewar flask *t* could be considered the same for all elements. The medium temperature was kept constant. If the specimen temperature *t* remained below the medium temperature θ (*t <* θ), then the specimen received heat *Q_a_* from the medium; if *t* > θ, then the specimen lost a certain amount of heat *Q_b_*. If the heating temperature interval *t*_2_ − *t*_1_ is chosen so that the equality *Q_a_ = Q_b_* is observed, then the balance of heat exchange with the medium becomes zero. Then, the additive heat capacity of the system can be determined using the following formula:

*C* = *Q*/(*t*_2_ − *t*_1_), (4)

where *Q* is heat released by the heater, which, according to Joule’s law, is equal to

*Q* = *IUτ*. (5)

For the equality *Q_a_ = Q_b_*, the times *τ*_1_ and *τ*_2_ and the corresponding temperatures *t*_1_ and *t*_2_ must be chosen so that the areas *F*_1_ and *F*_2_, replacing the value of the integral in Equation (3), are equal to *F*_1_ = *F*_2_ (see Figure 4).

#### 2.4.4. Calculation Reduction of the Semi-Adiabatic Calorimetric Test Results to the Isothermal Regime

Dewar flasks have some differences in the value of αS; thus, the same mix tested in different flasks shows a temperature curve that does not completely coincide temporally. This is due to the dependence of the hydration heat rate on temperature. To obtain comparable results, the heat of hydration *Q*, obtained using the semi-adiabatic method at an initial temperature of paste of 20 °C, was calculated for the isothermal hardening regime at a temperature of 20 °C using the reduced time hypothesis [41]. According to the hypothesis, at the moments of equal heat releases at *Q*_1_ = *Q*_2_, the ratio of hydration heat rate, as well as the corresponding periods *τ*_2_ and *τ*_1_, remains constant throughout the entire process.

(6) $$\frac{{\left(dQ/d\tau \right)}_{1}}{{\left(dQ/d\tau \right)}_{2}}=\frac{\tau_{2}}{\tau_{1}}=f_{t}=\mathrm{const}.$$

The temperature function *f_t_* was calculated using the following formula:

(7) $$f_{t}=2^{\frac{t_{1}-t_{2}}{\varepsilon }},$$

where *ε* is the characteristic temperature difference. If *t*_1_ − *t*_2_ = ε, then *f_t_* is equal to 2. That is, when the temperature rises by *ε* degrees, the heat of hydration rate increases twofold.

The value of *ε* is obtained from experimental data on the hydration heat of paste at (at least) three temperatures. It was established that the characteristic temperature difference *ε* is not constant, but depends on the temperature. This dependence is approximated by a linear function *ε* = *kt + l*, where *k* ≈ 0.13 and *l* ≈ 8; they are empirical process characteristics.

The reduced time for each value of hydration heat is calculated using the following formula:

*τ*_2_ = *τ*_1_ · *f_t_*. (8)

#### 2.4.5. Specimens 

Specimens from fly ash–sand paste of a cylindrical shape with a diameter of 62 mm and a height of 160 mm were used to determine the heat of hydration. The mixes of the specimens and their symbols are given in Table 5.

### 2.5. Compressive Strength

To determine the compressive strength, a PGM-50MG4 hydraulic press with a maximum force of 50 kN was used. During the test, the loading rate was maintained at 50 ± 10 kPa/s.

The compositions of the fly ash–sand mortar Q1–Q4 (see Table 5), tested for hydration, were also tested for compressive strength at the age of 28 days. Specimens of a cubic shape, in the amount of three pieces per test, had dimensions of 3.2 cm × 3.2 cm × 3.2 cm. The specimens were stored in a laboratory room with a temperature of 20 ± 2 °C for the first 7 days in covered forms and for the rest of the time in a desiccator above the water. Specimens before testing during visual inspection showed no signs of expansion and cracking.

## 3. Results

### 3.1. Evaluation of the Possibility of Regulating the Fly Ash Expansion Using Chemical Additives

**Figure 5:**
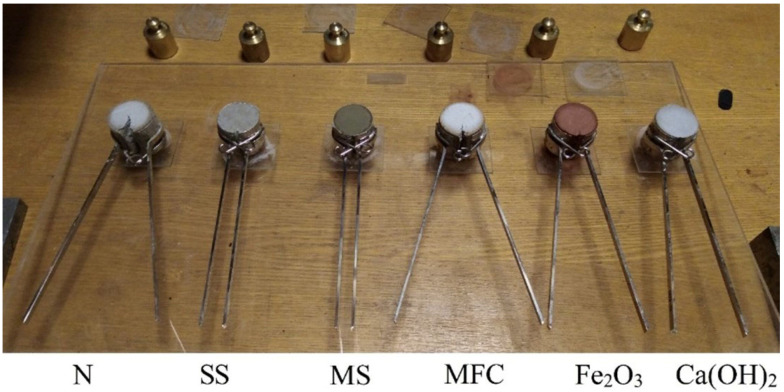
Photo of specimens after the expansion test (designations according to Table 4).

**Figure 6:**
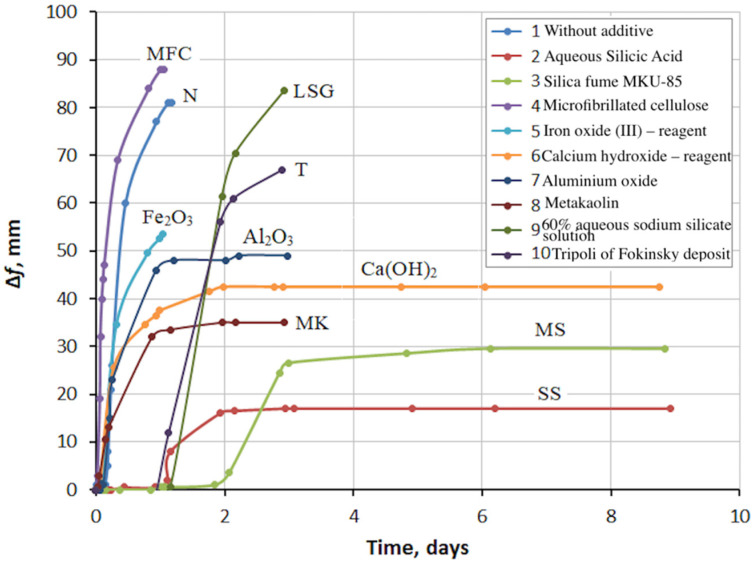
Effect of additives on fly ash expansion (designations according to Table 4).

The types of additives used and their content in the paste by weight of fly ash are presented in Table 4. A photograph of some specimens after the expansion test is shown in Figure 5. The effect of additives on the expansion kinetics of the fly ash is shown in Figure 6.

Figure 5 and Figure 6 show that the control mix N (fly ash + water) had a very high expansion. The experiment with this mix and two others was interrupted after about 1 day, in order to avoid damage to the Le Chatelier molds. It was expected that the addition of microcellulose (MFC) would retain expansion by reinforcing the paste, but this did not happen. On the contrary, the microcellulose sample expansion started earlier and was higher than the control mix (N) expansion. The addition of Fe_2_O_3_ somewhat reduced the expansion, possibly due to the partial binding of lime into calcium hydroferrite. The addition of Ca(OH)_2_ reduced expansion more significantly than Fe_2_O_3_. This can be explained using Le Chatelier’s principle, according to which an increase in the concentration of the reaction product, which is Ca(OH)_2_, inhibits the reaction. 

Mixes containing 60% aqueous sodium silicate solution (LSG) and tripoli (T) additives showed high expansion, not inferior to the control mix (N). However, unlike the control mix, these mixes had an expansion delay of about 1 day, which may indicate a neutralization reaction of some free lime. It should be noted that these substances also contained SiO_2_ in their composition. The presence of silica in metakaolin (Al_2_O_3_·2SiO_2_) contributed to a decrease in the final fly ash expansion compared to pure Al_2_O_3_. The behavior of the composition with metakaolin (MK) turned out to be very interesting. The volumetric expansion of the samples averaged 30.3% of the initial volume; however, no cracking was observed. In this case, the compressive strength was 9.9 MPa.

In the case of silica fume (MS) and silicic acid (SiO_2_∙nH_2_O) close in composition, it can be seen that the expansion did not begin immediately, but after some time. During this time, silica bound free lime into amorphous calcium hydrosilicate and inhibited its hydration. When all the silica was used up, the reaction of lime with water began, accompanied by expansion. The longer expansion delay period of the silica fume mix than with the silicic acid mix was due to the higher amount of silica fume required. However, silicic acid was more effective, as it bound more lime in less time. The final value of expansion for the mix with silicic acid turned out to be less than that for the mix with silica fume. Consequently, less lime was left free to react with water.

**Figure 7:**
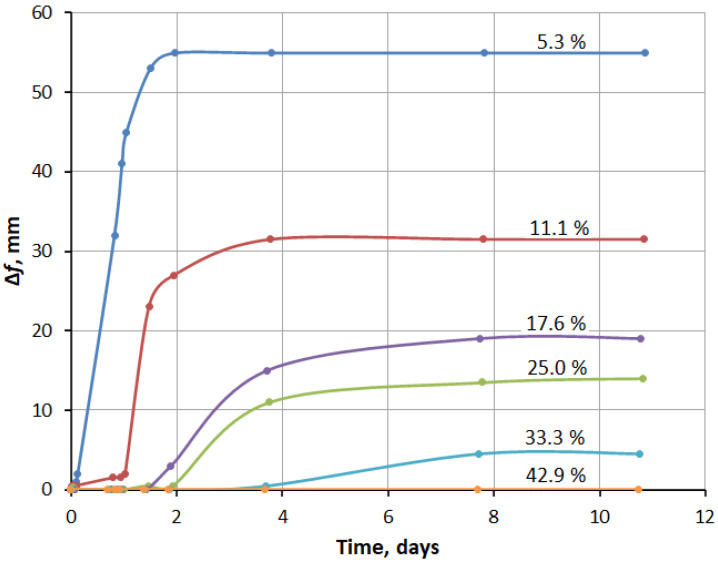
Effect of silica fume addition on fly ash expansion.

**Figure 8:**
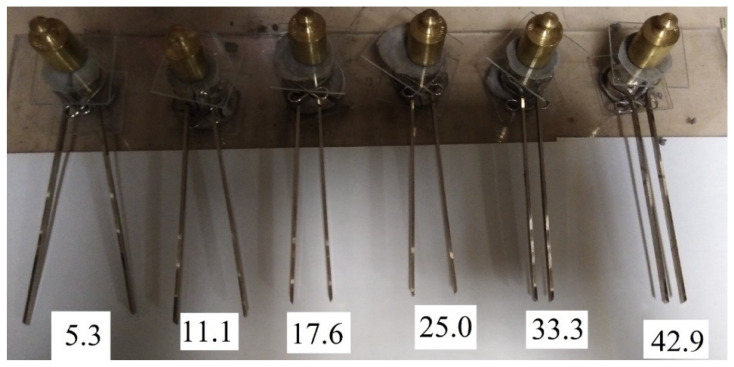
Expansion of specimens with content of silica fume additive from 5.3% to 42.9% by weight of fly ash after 4 days of air curing.

**Figure 9:**
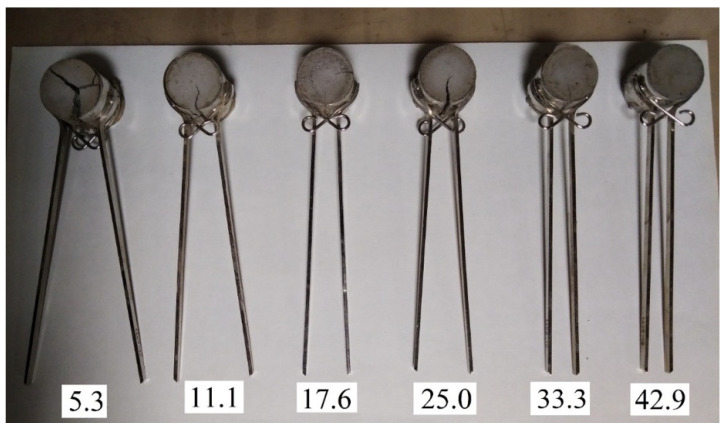
Expansion of specimens with content of silica fume additive from 5.3% to 42.9% by weight of fly ash after 11 days of air curing.

The delay period (incubation period) and the final expansion depend on the amount of silica fume. This was confirmed by the following experiment: from fly ash and silica fume with the same W/S = 0.42, six mixes were prepared, differing in the content of silica fume, as 5.3%, 11.1%, 17.6%, 25.0%, 33.3%, and 42.9% by weight of fly ash. These mixes were tested for expansion in Le Chatelier molds. The test results are shown in Figure 7, Figure 8 and Figure 9.

Figure 7, Figure 8 and Figure 9 show that the expansion processes of the specimens practically stopped on the fourth day of hardening.

The expansion curves (see Figure 7) include three characteristic sections: (1) the incubation period, which is longer with a greater silica fume content; (2) a period of intense strain growth, the duration of which increases with increasing silica fume content; (3) a period of stabilization, when further expansion stops.

Of the six mixes, only specimens with 42.9% silica fume retained the original volume and did not have cracks. In other cases, expansion and cracking increased with a decrease in the silica fume content. A similar effect of silica fume was found on mortar samples with fly ash [42]. On the other hand, the authors of [43] presented opposite results, which was explained by the effect of silica fume on the rate of ettringite formation.

**Figure 10:**
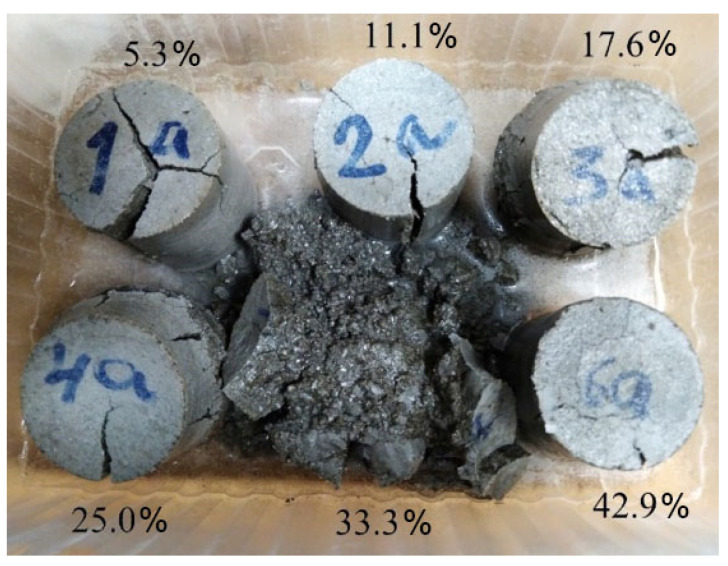
Expansion of specimens with content of silica fume additive from 5.3% to 42.9% by weight of fly ash after 11 days of air curing and 2 days of water curing.

After 11 days of hardening, when the expansion of all specimens stabilized, the samples were removed from the Le Chatelier molds and placed in water for 2 days, as a result of which the expansion resumed. Significant swelling of the specimens occurred. The specimens took a barrel shape and were divided into two parts due to a longitudinal crack. When trying to extract specimens from the water, they fell apart in the fingers. Here, an inverse dependence on the content of silica fume was observed, however, the mix with 33.3% silica fume, unlike other specimens, completely fell apart. Specimens containing 42.9% silica fume were the least affected (see Figure 10).

Thus, silica fume showed the greatest efficiency of all tested additives as an inhibitor of the high-calcium fly ash expansion. Silica fume at a content of 42.9% by weight of fly ash provided crack resistance and no expansion during air curing, but did not impart water resistance to the mix.

### 3.2. Test Results of X-ray Diffraction Analysis and Differential Thermal Analysis

**Figure 11:**
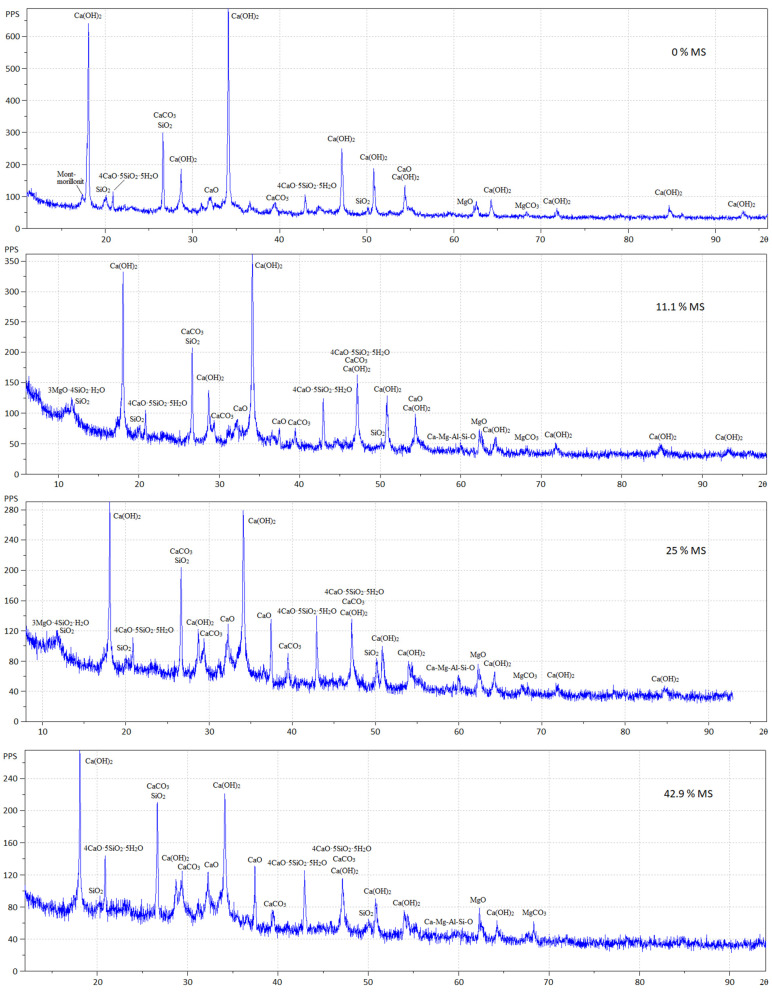
X-ray diffraction patterns of fly ash with silica fume addition (MS) in the amount of 0%, 11.1%, 25.0%, and 42.9 % by weight of fly ash.

The X-ray diffraction patterns of fly ash with silica fume addition (MS) after 11 days of air curing are shown in Figure 11.

Hydrated fly ash without additives contains crystallohydrates, mainly Ca(OH)_2_ and a small amount of calcium hydrosilicate of the composition 4CaO·5SiO_2_·5H_2_O, representing tobermorite.

Fly ash hydration products in the presence of 11.1% silica fume were represented by a lower content of calcium hydroxide and a larger amount of tobermorite. With a silica fume content of 25% and 42.9%, the hydrosilicates 1.5CaO·SiO_2_·nH_2_O and 2CaO·SiO_2_·nH_2_O were also identified in a small amount. In the presence of silica fume, X-ray diffraction patterns showed the presence of CaO_free_ in the samples. The ratio between Ca(OH)_2_, CaO_free_ and hydrosilicates depending on the content of silica fume is presented in Table 6.

Table 6 shows that CaO_free_ in the fly ash without additives was completely hydrated in 11 days. In [44], full hydration of free lime in expanding clinker was achieved in 7 days. Moreover, a small amount of tobermorite 4CaO·5SiO_2_·5H_2_O was formed as a result of hydration of 2CaO·SiO_2_ present in the fly ash. The presence of silica fume did not result in complete hydration of CaO_free_. Between 8.1% and 18.2% of CaO_free_ remained in the system. A greater silica fume content led to the formation of more hydrosilicates and a greater amount of unreacted free CaO_free_, with less Ca(OH)_2_ contained in the system. This circumstance explains why air-cured specimens without signs of expansion experienced swelling and cracking when placed in water. Apparently, there was a shortage of water for the complete slake of free lime with the introduction of silica fume, since, in this case, water was additionally spent on the formation of calcium hydrosilicates, the amount of which increased with increasing content of silica fume. There is a hypothesis [45] that expanding mixtures containing fly ash produce more ettringite than without fly ash; however, increasing the lime content in the fly ash slows down the rate of ettringite formation and promotes the formation of monosulfate [46,47]. In our case, no noticeable content of ettringite was found in the system. Accordingly, it can be concluded that the expansion of the tested fly ash occurred as a result of the formation of crystalline Ca(OH)_2_. This was also confirmed in [48].

**Figure 12:**
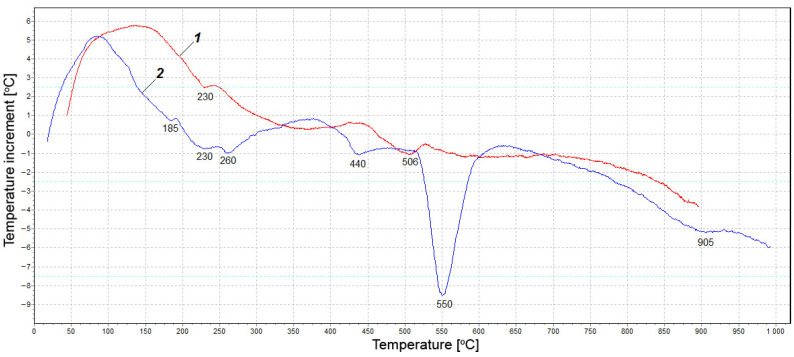
DTA curves for fly ash before (**1**) and after (**2**) hydration.

The results of X-ray diffraction analysis were confirmed by differential thermal analysis. Figure 12 presents the DTA curves for fly ash from Berezovskaya GRES before (curve 1) and after (curve 2) hydration for 11 days.

**Figure 13:**
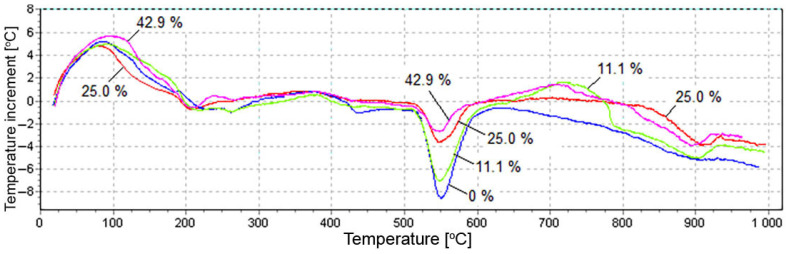
DTA curves for fly ash with different content of silica fume.

Thermogram 1 shows no significant thermal effects. Small endothermic peaks at 230 °C and 506 °C can be respectively attributed to the loss of water of hydrosilicate and calcium hydroxide partially hydrated during storage. A deep endothermic effect at 550 °C (see Figure 13) indicates that a large amount of Ca(OH)_2_ was formed as a result of free lime hydration. A wide endothermic valley in the range of 150–370 °C with separate small effects was probably associated with the loss of adsorption water by various hydrates. The endothermic effect at 440 °C presumably corresponds to the dehydration of calcium hydrosilicate. The narrow valley at 905 °C was highly likely related to lime decarbonization. The thermograms shown in Figure 13 characterize the effect of silica fume on the hydration of fly ash from Berezovskaya GRES.

Figure 13 shows that, when the silica fume content increased, the depth of the endothermic peak at 550 °C decreased. This was due to the decomposition of Ca(OH)_2_, confirming the XRD data obtained above. The decrease in the calcium hydroxide content is associated with three factors. First, the rate of lime hydration is drastically slowed down in the presence of silica fume, and unreacted free lime remains in the system for a long time, as shown below from the exothermic study. Secondly, a reaction occurs between Ca(OH)_2_ and SiO_2_ with the formation of a small amount of calcium hydrosilicates, as evidenced by insignificant effects on thermograms in the range of 150–270 °C, associated with the dehydration of silicates. Thirdly, endothermic peaks in the range of 890–920°C indicate the decomposition of calcium carbonate formed as a result of carbonization of Ca(OH)_2_ with air carbon dioxide during the hardening of the samples.

The kinetics of the reaction injvolving silica fume, fly ash free lime, and water can be followed from the heat release curves of the mixture during hydration.

### 3.3. Heat of Hydration Test Results 

The heat of hydration of high-calcium fly ash was determined using a mix with standard polyfractional sand. Three specimens of each mix were tested. The multichannel meter registrar “Terem-4” recorded thermocouple readings every 30 min. Four mixes of fly ash–sand paste (Q1–Q4) with the same amount of fly ash and different silica fume content of 0%, 11.1%, 25.0%, and 42.9% by weight of fly ash were tested (Table 5).

**Figure 14:**
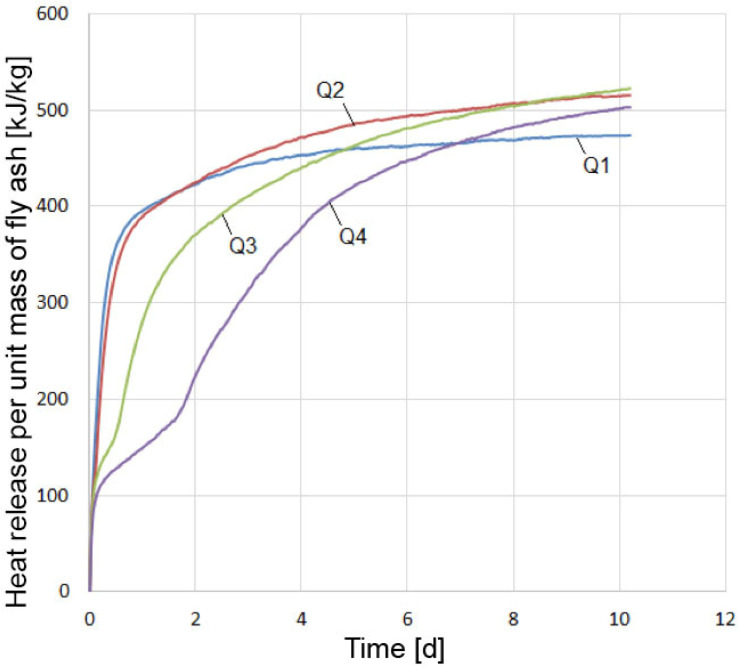
Influence of silica fume content on mortar heat release per unit mass of fly ash at a temperature of 20 °C: Q1 is without additive; Q2 is with 11.1% additive; Q3 is with 25% additive; Q4 is with 42.9% additive.

The mortar heat release per unit mass of fly ash at a temperature of 20 °C, depending on the silica fume content, is shown in Figure 14.

Pure fly ash (curve Q1) reacted very violently with water due to the slaking of free lime. Approximately 1 day after intense heat release, the process slowed down sharply and came to an end by the fourth day. This is consistent with the results of [44], where more than 50% CaO_free_ reacted on day 1, and almost 100% CaO_free_ reacted by day 7. In the presence of 11.1% silica fume (curve Q2), the reaction rate in the initial period up to 1 day was slightly inferior to that of pure fly ash, but began to exceed the latter; by 10 days, the heat release was approximately 9% higher than that of mix Q1. In the case of 25% and, even more so, 42.9% of silica fume content, a sharp slowdown in the process of heat release and, consequently, in the reaction of lime slaking was observed. On the 11th day, free lime still remained unslaked in the system, as seen from the XRD results. However, the reaction rate further increased, and, by the end of the experiment, it was higher than that of mix Q2. A higher silica fume content in the mix resulted in a lower heat release rate in the initial period of hardening (up to 0.5–1.5 days) and a higher rate in the future.

**Figure 15:**
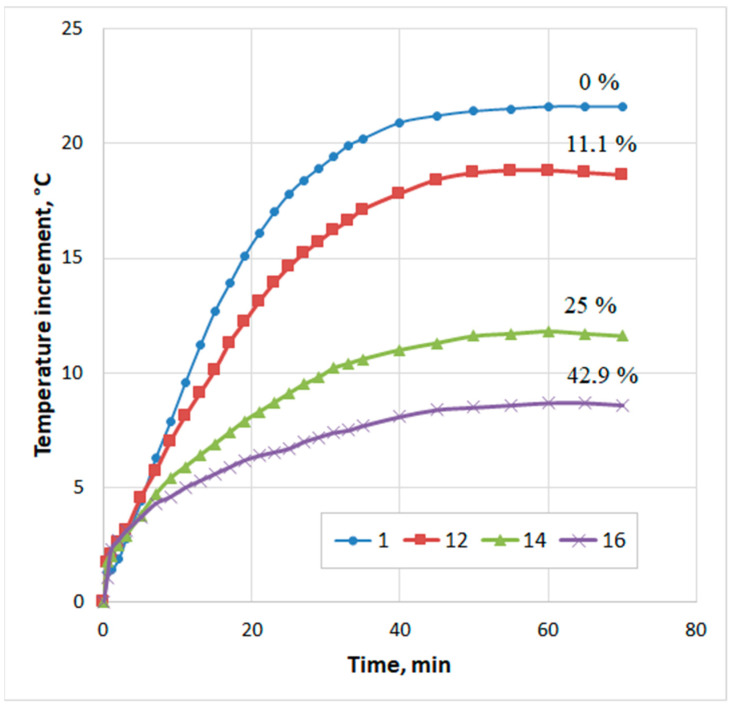
Influence of silica fume content on temperature increase of fly ash–silica fume paste (designations according to Table 4).

Heat of hydration experiments require a certain amount of time to prepare the mix, prepare the specimens, place the specimens in the Dewar flask, etc. In our case, this time was about 30 min. During this period, intense heat of hydration occurs; however, it is not recorded in the experiment. Therefore, additional tests were performed to determine this heat of hydration. First, 30 g of a thoroughly mixed dry composition of fly ash and silica fume was placed on the bottom of the Dewar flask. A closed container of water suspended on a thin thread was placed over this mixture in the Dewar flask. The water amount was taken from the calculation of W/S = 0.65. The closed Dewar flask and a glass thermometer with a division value of 0.1 °C were kept for 1 day in a room with a constant temperature. Before the start of the experiment, the temperature of the dry mix in the Dewar flask was measured and taken as the initial temperature of the sample. The dry mix in the Dewar flask was closed with prepared water and quickly stirred with the end of a thermometer, fixing the temperature. The first reading was taken after 30 s of the addition of water to the mix. Each mix was tested twice, and the average value was taken. The results of measuring the temperature of the fly ash–silica paste are shown in Figure 15. The figure shows that, with an increase in the silica fume content, the rate of lime hydration dropped sharply, starting from the first moment of the reaction. Here, the same pattern of silica fume influence was observed as in the initial period of hardening of the fly ash–sand mix (Figure 14). Such an effect of silica fume can be explained by a decrease in the transfer rate in solutions [44].

### 3.4. Compressive Strength Test Results

**Figure 16:**
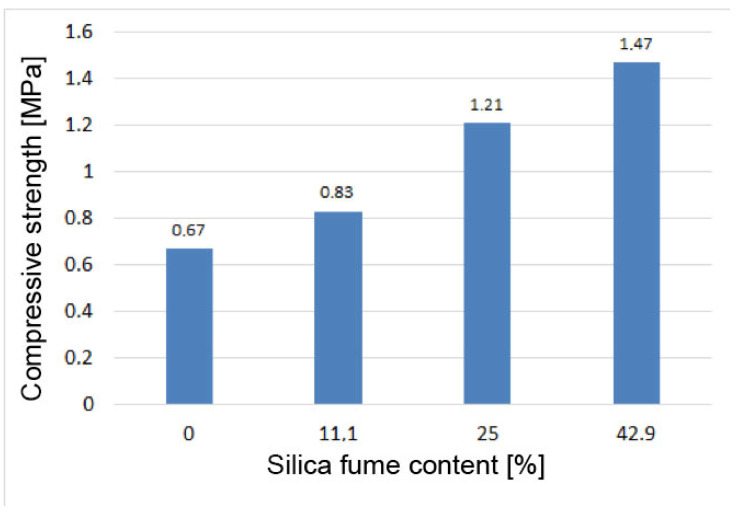
Compressive strength of specimens of fly ash–sand paste Q1–Q4.

The results of determining the compressive strength of the samples are shown in Figure 16.

Figure 16 shows that the strength of the specimens increased with increasing silica fume content. On average, for every percentage of silica fume introduced, the compressive strength increased by 2.7%. The increase in fly ash strength when 42.9% silica fume was added to mix was 119%, i.e., more than twofold. The low strength of the tested fly ash–sand paste should be noted; however, it must be borne in mind that the binder/sand ratio in these mixes ranged from 1:5 to 1:7.

On the basis of preliminary data not published here, it was established that the strength and water resistance of mixes with fly ash from Berezovskaya GRES can be increased by adding some accelerating agents.

## 4. Conclusions

1. The fly ash of Berezovskaya GRES, captured by electrostatic precipitators, is capable of self-setting and hardening due to its chemical composition. However, the hardening of Beryozovskaya fly ash is accompanied by strong expansion and cracking due to the large amount of free lime. Therefore, this fly ash is not used as a binder or as a concrete additive and is also not suitable for granulation.
2. A search for additives to the fly ash, which could prevent the expansion, was carried out. Aqueous silicic acid SiO_2_∙nH_2_O, silica fume MKU-85, micro-fibrillated cellulose, Fe_2_O_3_ (reagent, pure), calcium hydroxide Ca(OH)_2_ (reagent, pure), Al_2_O_3_ (AR), metakaolin Al_2_O_3_·2SiO_2_, 60% aqueous sodium silicate solution Na_2_O(SiO_2_)_n_, and tripoli of the Fokinsky deposit were tested in the Le Chatelier mold. The greatest effect was obtained from silicic acid and silica fume, and the smallest effect was obtained from 60% aqueous sodium silicate solution, Tripoli, and microcellulose.
3. The expansion and cracking of the fly ash could be completely prevented by the addition of silica fume in an amount of about 40% by weight of the fly ash. At lower silica fume values, expansion was observed. The expansion increased with a decrease in the silica fume content.
4. Silica fume allowed the fly ash to harden normally and gain strength in air, but did not provide sufficient water resistance to the hardened paste. Specimens swelled and cracked when placed in water.
5. The results of XRD and DTA showed that free lime in the fly ash was completely hydrated in 11 days in the absence of silica and gave the highest degree of expansion. In the presence of silica fume, the hydration of lime by the specified time was not complete, and the expansion was reduced. Unslaked free lime remained in the system. Its amount increased with an increase in the silica fume content, while the content of Ca(OH)_2_ decreased. This circumstance could explain the low water resistance by the lack of water for the complete redemption of free lime, since water is additionally spent on the formation of calcium hydrosilicates, the amount of which increases with an increase in the dosage of silica fume. However, exothermic data showed that silica fume inhibited CaO hydration from the first seconds of the reaction.
6. This work is a preliminary assessment of the effectiveness of silica fume for Berezovskaya GRES fly ash in relation to its expansion during hydration, due to the high content of free lime. On the basis of some data not included in the article, it was established that the strength and water resistance of mixes with fly ash from Berezovskaya GRES and silica fume can be increased by adding some accelerating agents.

## Figures and Tables

**Table 1:** Average composition of fly ash.

SiO_2_	Al_2_O_3_	FeO	Fe_2_O_3_	MgO	CaO	SO_3_	K_2_O	Na_2_O	CO_2_
15.8 ± 6.9	8.0 ± 0.2	0.7 ± 1.0	7.0 ± 1.7	4.9 ± 0.8	46.7 ± 5.4	7.2 ± 4.8	0.76 ± 0.4	1.28 ± 0.9	3.61 ± 1.88

**Table 2:** Physical properties of fly ash.

Physical Property	Unit Measure	Value
Residue on sieve No. 008	%	8.7
Residue on sieve No. 005	%	16.7
Bulk density	g/cm^3^	1094
True density	g/cm^3^	3.09
Specific surface area (Blaine)	cm^2^/g	2815
Volume median diameter	µm	7.7

**Table 3:** Phase composition of fly ash from Berezovskaya GRES.

Chemical Compound	Conditional Content (%)	Chemical Compound	Conditional Content (%)
CaO	42.6	2CaO·Al_2_O_3_	3.4
SiO_2_	9.7	4CaO·Al_2_O_3_· Fe_2_O_3_	0.9
Al_2_O_3_	5.4	2CaO·0.7Al_2_O_3_·0.3Fe_2_O_3_	0.7
MgO	4.6	4CaO·2MgO·Al_2_O_3_·Fe_2_O_3_	2.2
2CaO·SiO_2_	5.6	2CaO·0.2MgO·0.5Al_2_O_3_·0.3Fe_2_O_3_·0.2SiO_2_	3.3
3CaO·MgO·2SiO_2_	2.6	3Al_2_O_3_·2SiO_2_	2.6
CaO·2Al_2_O_3_	4.2	TiO_2_	1.7
3CaO·Al_2_O_3_	2.8	C	7.7

**Table 4:** Types of additives in experiments on expansion of fly ash paste in Le Chatelier molds.

Mix Number	Additive Type	Mix Symbol	Additive Content by Weight of Fly Ash (%)	Water–Solid Ratio W/S
1	Without additive	N	-	0.43
2	Aqueous silicic acid, SiO_2_∙nH_2_O—AR	SS	18.4	0.8
3	Silica fume MKU-85	MS	20.0	0.42
4	Micro-fibrillated cellulose	MFC	32.0	0.39
5	Iron oxide Fe_2_O_3_—reagent, pure	Fe_2_O_3_	20.0	0.35
6	Calcium hydroxide Ca(OH)_2_—reagent, pure	Ca(OH)_2_	17.4	0.33
7	Al_2_O_3_—AR	Al_2_O_3_	40.0	0.48
8	Metakaolin Al_2_O_3_·2SiO_2_	MK	42.9	0.5
9	60% aqueous sodium silicate solution Na_2_O(SiO_2_)_n_	LSG	6.6	0.51
10	Tripoli of the Fokinsky deposit (Bryansk region, Russia)	T	42.3	0.54
11	Silica Fume MKU-85	MS	5.3	0.42
12	Silica Fume MKU-85	MS	11.1	0.42
13	Silica Fume MKU-85	MS	17.6	0.42
14	Silica Fume MKU-85	MS	25.0	0.42
15	Silica Fume MKU-85	MS	33.3	0.42
16	Silica Fume MKU-85	MS	42.9	0.42

**Table 5:** Material consumption of fly ash–sand pastes.

Material	Material Consumption (kg/m^3^)			
	Q1	Q2	Q3	Q4
Fly ash from Berezovskaya GRES	210	210	210	210
Silica fume MKU-85	0	23 (11.1%)	53 (25%)	90 (42.9%)
Polyfractional sand	1645	1623	1558	1476
Water	318	316	327	340
Total	2173	2172	2147	2116

**Table 6:** Crystalline phases of fly ash.

Sample Type	Phase Content (%)		
	Ca(OH)_2_	CaO_free_	Hydrosilicates
Fly ash original	-	100	-
Fly ash after hydration	96.2	-	3.8
Fly ash + 11.1% silica fume after hydration	80.1	8.1	11.8
Fly ash + 25% silica fume after hydration	67.8	17.9	14.3
Fly ash + 42.8% silica fume after hydration	63.3	18.2	18.6

## Data Availability

Not applicable.
